# Limitations in Physical Activities and Loneliness: The Role of Guilt for Perceiving Oneself as a Burden

**DOI:** 10.1093/geroni/igab046.1763

**Published:** 2021-12-17

**Authors:** María del Sequeros Pedroso Chaparro, Isabel Cabrera, Carlos Vara-García, José Adrián Fernandes-Pires, Samara Barrera-Caballero, Laura Mérida-Herrera, María Márquez-González, Andrés Losada-Baltar

**Affiliations:** 1 Universidad Autónoma de Madrid, Madrid, Madrid, Spain; 2 Universidad Rey Juan Carlos, Madrid, Madrid, Spain; 3 Rey Juan Carlos University, Alcorcón, Madrid, Spain; 4 Rey Juan Carlos University, Alcorcón, Madrid, Madrid, Spain

## Abstract

Loneliness is a prevalent problem associated with negative health consequences for older adults, such as greater cognitive decline. Limitations to perform physical activities have been associated with greater loneliness in older adults. This association could be moderated by maladaptive social cognition or feelings, such as guilt associated with perceiving oneself as a burden. The objective of this study was to analyze the moderating effect of guilt associated with perceiving oneself as a burden in the relationship between limitations in physical activities and loneliness. Participants were 195 community-dwelling people 60 years or older not showing explicit cognitive or functional limitations that prevent activities of daily life, but who may present limitations in some physical activities (e.g., walking a kilometer or more). A linear regression analysis was conducted for testing the interaction between limitations in physical activities and guilt for perceiving oneself as a burden in loneliness, controlling for gender and age. The interaction between limitations in physical activities and guilt for perceiving oneself as a burden was the only significant predictor of loneliness and the model explained 18.30% of the variance. Limitations in physical activities influenced loneliness when people have high levels of guilt for perceiving oneself as a burden. This study suggests that guilt for perceiving oneself as a burden may play an important role in the association between limitations in some physical activities and loneliness.

